# The promise of *N*‐acetylcysteine in the treatment of obsessive‐compulsive disorder

**DOI:** 10.1111/cns.14653

**Published:** 2024-02-22

**Authors:** Massimo Carollo, Nicola Carollo, Giulia Montan

**Affiliations:** ^1^ Department of Diagnostics and Public Health University of Verona Verona Italy; ^2^ Department of Medicine University of Verona Verona Italy; ^3^ Department of Women's and Children's Health University of Padova Padova Italy

**Keywords:** compulsive behavior, glutamic acid, *N*‐acetylcysteine, obsessive behavior, obsessive‐compulsive disorder

This letter to the Journal is intended to raise awareness within the scientific community about the potential use of *N*‐acetylcysteine (NAC) as an alternative treatment for obsessive‐compulsive disorder (OCD), primarily targeting glutamatergic system dysfunctions and modulating inflammatory cytokines.^1^

Obsessive‐compulsive disorder is a debilitating psychiatric condition characterized by persistent, intrusive thoughts (obsessions) and repetitive behaviors or mental acts (compulsions). The lifetime prevalence of OCD is estimated at 2%–3%, a figure that may be underestimated due to its frequent misdiagnosis.^1^, ^2^ Current first‐line treatments include selective serotonin reuptake inhibitors (SSRIs) and cognitive‐behavioral therapy (CBT).^3^ However, a significant portion of patients remains refractory to these treatments, necessitating the exploration of alternative therapeutic strategies. Some studies explored the potential benefits of some glutamate‐modulating agents such as riluzole, memantine, NAC, D‐cycloserine, and ketamine.^1^

Dysregulation in the glutamatergic system, the primary excitatory neurotransmitter system in the human brain, is implicated in the pathophysiology of OCD. Indeed, the glutamatergic system is crucial for neuroplasticity, learning, and memory processes, and recent research has uncovered several glutamatergic abnormalities in individuals with OCD. One important finding is the altered levels of glutamate observed in specific brain regions, such as the anterior cingulate cortex.^4^ This area is critical for attention allocation and emotion regulation, and their dysregulation may contribute to OCD symptoms. The regulation of glutamate homeostasis presents a multifaceted challenge, as glutamate has the capability to diffuse beyond the confines of the synaptic cleft. While the stimulation of N‐methyl‐D‐aspartate (NMDA) receptors located postsynaptically facilitates the conveyance of information, synaptic plasticity, and trophic effects on neuronal cells, the triggering of NMDA receptors situated outside the synapse inhibits these functions and may precipitate excitotoxicity, resulting in neuronal damage and apoptosis.^4^, ^5^ Beyond glutamate levels, the transport of glutamate also appears to be affected in OCD. Glutamate transporters, which are responsible for clearing glutamate from the synaptic space to prevent excitotoxicity, may function abnormally in OCD, leading to an imbalance in excitatory signaling.^6^ Additionally, alterations in NMDA and α‐amino‐3‐hydroxy‐5‐methyl‐4‐isoxazolepropionic acid (AMPA) receptors, and intracellular signaling pathways modulated by glutamate, have been implicated in OCD, further supporting the notion of glutamatergic dysregulation.^5^, ^6^

An interesting candidate to target and modulate the glutamatergic system is NAC, a derivative of the amino acid cysteine. In vivo, NAC donates a cysteine unit that is integral to the synthesis of glutathione. Cysteine that is not utilized in this process crosses the blood–brain barrier, facilitated by sodium‐dependent transport pathways. Once within the central nervous system, it is oxidized into cystine.^7^ This cystine subsequently gets exchanged by astrocytes with glutamate, which is released into the extracellular space, through a cystine‐glutamate antiporter, leading to the activation of inhibitory metabotropic glutamate receptors mGLuR2/3 on glutamatergic nerve terminals, resulting in the reduction of synaptic release of glutamate.^5^, ^8^ Additionally, preclinical studies have shown glutathione to potentiate brain *NMDA receptor* response to glutamate.^9^ Therefore, changes in the levels of neuronal glutathione may not only alter available glutamate levels but also have direct consequences on glutamatergic function.

It's worth mentioning that NAC may have other biological functions in the brain hypothesized to be implicated in OCD, such as the modulation of dopamine release and the reduction in inflammatory cytokine formation. These properties, along with the reduction of oxidative stress and the re‐establishment of glutamatergic balance, would lead to an increase in growth factors, such as brain‐derived neurotrophic factor, and the regulation of neuronal cell death through B‐cell lymphoma 2 expression.^5^, ^7^

To the best of our knowledge, only five randomized controlled trials have tested the potential efficacy of NAC as an adjunctive treatment in OCD, four of which reported significant reductions in Yale‐Brown Obsessive‐Compulsive Scale (Y‐BOCS) scores at dosages of 2000–3000 mg/day.^10^, ^11^, ^12^, ^13^, ^14^, ^15^

There are multiple limitations in using NAC for treating OCD. First, while these pivotal studies have been excellent in exploring its therapeutic potential, the rationale behind the dosages used in those studies is not clear. There is a need for further research with various dosages, including higher ones, and over longer periods, which are often necessary to observe improvements in various psychiatric disorders, including OCD. Second, safety concerns. Notably, NAC has proved to be safe even in very high dosages (e.g., in clinical toxicology).^7^ Potential and very rare side effects with the oral administration include gastrointestinal disturbances and hypersensitivity reactions such as anaphylactic shock, anaphylactic/anaphylactoid reactions, bronchospasm, angioedema, rash, and itching.^7^, ^8^ These side effects might not be attributable to NAC itself, but rather to other excipients in the formulations, such as sodium benzoate, parahydroxybenzoates, sorbitol, aspartame, Sunset Yellow FCF (E110), lactose, and propylene glycol. However, safety data on long‐term use are missing. Lastly, the pharmaceutical forms of NAC available on the market typically contain 200–600 mg. The Summary of Product Characteristics recommending the maximum dosage of 600 mg and the presence of other excipients restricts the use of higher doses of NAC that may be needed to reach therapeutic levels for OCD.^16^

In conclusion, despite the captivating evidence associating glutamatergic abnormalities with OCD, the nature of this relationship remains complex and not yet fully understood. OCD likely emerges from a multifaceted interplay of factors, encompassing neurochemical imbalances, genetic predispositions, environmental triggers, and psychological influences. However, the use of NAC as an augmentation therapy to CBT or SSRIs may be not only effective but also safe, and further research, including more rigorous, large‐scale trials, is needed to establish efficacy, optimal dosages, and long‐term outcomes. To date, empirical and off‐label use is limited by the absence of medicinal formulations with the appropriate dosage specifically for OCD treatment.

## AUTHOR CONTRIBUTIONS

M.C. supervised the study and conceived the concept. M.C. wrote the first draft of the manuscript. M.C., N.C., and G.M. contributed to the literature search and critically revised the work. All authors have made important intellectual contributions and have seen and approved the manuscript for submission. The corresponding author (M.C.) attests that all listed authors meet authorship criteria and that no others meeting the criteria have been omitted.

## FUNDING INFORMATION

This contribution was made possible by the extraordinary fund for Open Access publication of the University of Verona.

## CONFLICT OF INTEREST STATEMENT

None.

## Data Availability

Data sharing not applicable to this article as no datasets were generated or analysed during the current study.
